# Prospective results of concurrent radiation therapy and weekly paclitaxel as salvage therapy for unresectable locoregionally recurrent breast cancer

**DOI:** 10.1186/s13014-019-1321-1

**Published:** 2019-07-02

**Authors:** Gang Cai, Lu Cao, Youlia M. Kirova, Yan Feng, Jia-Yi Chen

**Affiliations:** 1 Department of Radiation Oncology, Shanghai Cancer Center, Shanghai Medical College, Fudan University, Shanghai, China; 2 Department of Radiation Oncology, Ruijin Hospital, Shanghai Jiao Tong University, Shanghai, China; 3 Department of Radiation Oncology, Institut Curie, Paris, France

**Keywords:** Locoregional recurrent breast cancer, Radiotherapy Paclitaxel, Concurrent chemoradiation

## Abstract

**Purpose:**

To investigate the efficacy and toxicity of concurrent radiation therapy (RT) and paclitaxel in the treatment of unresectable locoregionally recurrent breast cancer (RBC) after mastectomy as primary treatment.

**Methods:**

This was a prospective monocentric study of 51 patients (pts) diagnosed with unresectable locoregionally RBC after mastectomy and treated between 2008 and 2012. Radiotherapy (RT) was delivered at 60 Gy in 30 treatment fractions to recurrent sites. Chemotherapy was weekly paclitaxel of 50 mg/m^2^ for 5 weeks. All pts. underwent clinical examination, CT or PET/CT every 3 months in first 2 years and then every 6 months. Tumor response was evaluated clinically and by CT using the RECIST criteria. Toxicity was assessed weekly during RT by the NCI common toxicity criteria (version 3.0).

**Results:**

Fifty-one pts. with 61 recurrent sites were studied. The median age was 49 years. Sites of RBC were chest wall in 20 patients (32.8%), supraclavicular in 19 (31.1%), axilla in 11 (18.0%), and internal mammary lymph nodes in the remaining 11 (18.0%). RBC presented as single in 25 pts., multiple in 20 pts. and diffuse growth in 6 pts. Clinical response was observed in 47 pts. (92.2%), with 36 (70.6%) complete and 11 (21.6%) partial responses. Two patients (3.9%) presented with stable disease and 2 progressive disease. The cumulative local progression-free survival rate was 62.8% at 2 year and 53.0% at 5 years after treatment. No grade 4 toxicity was observed. Grade 3 radiation dermatitis and leukocytopenia were observed in 10 (19.6%) and 12 (23.5%) pts., respectively. One patient experienced grade 2 pneumonitis.

**Conclusions:**

Concurrent RT and weekly paclitaxel could be an effective therapeutic option for unresectable locoregionally recurrent breast cancer after mastectomy with an acceptable toxicity profile.

## Introduction

Despite the increasing use of breast-conserving surgery, mastectomy with axillary lymph node dissection retains important in primary breast treatment. This treatment must be followed by radiation therapy in cases of lymph node involvement, as well as some cases of large tumors, lymphovascular invasion (LVI) and other factors of risk [1]***.*** Approximately 5–30% of patients treated with (modified) radical mastectomy develop locoregional recurrence (LRR) [1]. The salvage therapy of isolated LRR after mastectomy remains a difficult and challenging problem.

Postmastectomy patients with LRR are generally treated with combined-modality therapy, including surgery, radiotherapy, and systemic therapy. The excision of a recurrent tumor is a good local salvage therapeutic option [2]. However, the rate of second local failure after local excision alone is very high [3, 4], and some patients cannot undergo curative surgery because of locally unresectable disease, medical unfitness or an unwilling to accept the associated complications. RT is an effective method to combine with surgical excision to reduce the rate of second local failure and treat the inoperative isolated LRR with curative intent [4–6]. The combination of radiotherapy and chemotherapy has effect in improving the clinical outcome of patients with recurrent breast cancer [7]. However, the optimal regimens for concurrent chemoradiation (CRT) are not clearly defined. Paclitaxel has been considered a very effective cytotoxic drug for the treatment of systemic treatment of breast cancer [8]. Preclinical studies have demonstrated a synergistic effect of paclitaxel combined with RT and suggested radiosensitizing activity of paclitaxel [9]. Some studies have evaluated paclitaxel combined with RT and have demonstrated its safety and efficacy for the treatment of locally advanced breast cancer [10–12]. However, to the best of our knowledge, no previous prospective report has evaluated the efficacy and safety of weekly paclitaxel concurrent with RT for the treatment of unresectable locoregionally recurrent breast cancer after mastectomy. Based on these considerations, we carried out a prospective study to investigate the local effect and acute toxicity of paclitaxel concurrent with RT in patients with unresectable LRR of breast cancer.

## Materials and methods

### Eligibility criteria and exclusion criteria

Study participants were women with unresectable LRR of breast cancer after (modified) radical mastectomy who were seen at our hospital from January 2008 to June 2012. This prospective study was approved by our institutional review board, and all patients signed informed consents.

To be eligible, each patient had a histologically proven primary breast adenocarcinoma and cytologically or histologically proven recurrences. All patients were discussed by our multidisciplinary team before treatment and were diagnosed with LRR by histological confirmation. There was no previous treatment of locoregional RT (chest wall, supraclavicular or axillar regions) and no previous chemotherapy with paclitaxel in the last 6 months. There were measurable lesions in the fields of RT. Other inclusion criteria were as follows: age between 18 and 70 years; Eastern Cooperative Oncology Group performance score ≤ 1; adequate hematological, liver function, and other laboratory parameters (leucocytes > 4.0 × 10^9^/L, platelets > 100 × 10^9^/L, bilirubin < 1.5 the upper limit of normal range (ULN), aspartate aminotransferase/alanine aminotransferase ≤2.5 × ULN, and serum creatinine < 1.25 × ULN.

The exclusion criteria were as follows: metastatic breast cancer; prior RT of chest wall, supraclavicular or axillar regions; prior chemotherapy with paclitaxel in the last 6 months.; any other malignancy; a history or symptoms of connective tissue disorders (e.g. scleroderma or systemic lupus erythematosus); concurrent medical conditions that might preclude CRT, such as serious uncontrolled infection, significant coronary or cardiac conditions, steroid-dependent reactive airways diseases, or uncontrolled hypertension or diabetes; a psychiatric disorder; and pregnancy or lack of contraception in women with child-bearing potential.

### Radiation therapy

RT was delivered with a linear accelerator using a Med-Tec 350 breast board (Med-Tec Corporation, Orange, IA, USA) with both arms raised above their heads. All patients had a planning CT scan in the treatment position. CT images were acquired with 5-mm-thick intervals from the level of mandible through the lung base using a Philips Big Core CT scanner (Philips Medical Madison, Fitchburg, WI, USA). All CT images were exported to the Pinnacle treatment planning system (Philips Radiation Oncology Systems, Pinnacle version 8.0, Milpitas, CA) for contouring the target volumes and the nearby organs at risk and treatment planning. Intensity-modulated radiation therapy (IMRT) planning was used for each patient based on the planning CT. Target definition followed the recommendations of the ICRU reports No.83 [13]. The gross tumor volume (GTV) was determined by a combination of findings on physical exam, CT, and/or PET-CT. The clinical target volume (CTV) included the GTV, the ipsilateral chest wall (CW), supra- and infraclavicular regions (SC&IC), axillary lymph node (Ax) (if recurrent lesion extended to axillary lymph node), cervical lymph node (if recurrent lesion extended to supraclavicular lymph node) and intramammary lymph node (IM) (if recurrent lesion extended to intramammary lymph node). CTV was delineated according to the breast cancer atlas for radiation therapy planning consensus definitions of the Radiation Therapy Oncology Group (RTOG) (available at: http://www.rtog.org/CoreLab/ContouringAtlases/BreastCancerAtlas.aspx). PTV1 was generated with a 1 cm asymmetrical margin around the CTV. For dosimetric reporting, the PTV_EVAL was generated from the PTV1 by cropping the skin edge and excluding the chest wall. PTV2, within the limit of PTV1, was generated by expanding GTV by 2 cm. Bolus was used on the chest wall in PTV1 in each case and PTV2 in cases of skin involvement. The ipsilateral and contralateral lung, spinal cord, thyroid, humeral head, and heart were outlined as organs at risk. The use of 6 MV photons and/or electrons of appropriate energy and field arrangement was at the discretion of the treating oncologist. RT was planned up to 60 Gy in 30 treatment fractions: 46 Gy in 23 fractions to PTV1 and 14 Gy in 7 fractions to PTV2. The tolerances of normal tissues were defined as follows: ipsilateral lung, mean dose < 15 Gy, not > 30% received a dose > 20 Gy (V20 < 30%); contralateral lung, not > 10% received a dose > 5 Gy (V5 < 10%); spinal cord, maximum dose < 45 Gy; humeral head, mean dose < 25 Gy; heart, mean dose < 8 Gy for left-side lesions and < 2 Gy for right-sided lesions.

### Chemotherapy

Weekly paclitaxel was delivered concurrently with RT. Starting at day 1 of RT, patients received weekly paclitaxel of 50 mg/m^2^ for five consecutive weeks (days 1, 8, 15, 22 and 29).

If hematological toxicity developed (leucocytes < 3.0 × 10^9^/L or platelets < 100 × 10^9^/L), paclitaxel administration was delayed for a few days until counts recovered. If grade 3 non-hematological toxicity developed, paclitaxel administration was suspended until resolution to grade 2.

### Maintenance treatment

After concurrent CRT, maintenance treatment was recommended for all patients in need but was not included in the study protocol. Maintenance treatment (chemotherapy, hormone therapy, targeted therapy or surgery) for the patients was individualized, with no specific recommendations.

### Study design and data evaluation

The primary endpoint of this phase II study was local response after concurrent RT and paclitaxel regimen in patients with unresectable LRR of breast cancer. Secondary endpoints included acute toxicity and follow-up.The patients were routinely followed with clinical examinations, CT or PET/CT every 3 months for the first 2 years and every 6 months thereafter. Tumor response was evaluated using clinical examinations and CT according to the RECIST criteria at 3 months after concurrent CRT. Adverse events were assessed at least weekly during RT by the National Cancer Institute (NCI) common toxicity criteria (version 3.0).

Sample size estimation was performed based on the Simon’s optimal two-stage design [14, 15]. We hypothesized that the local response rate could be increased from 84% [16] to 95%. With a unilateral alpha error of 5% and a statistical power of 80%, a planned of 46 subjects were needed. With an anticipated drop-out rate of 10%, a total of 51 subjects were finally necessary.

The different parameters of each individual patient were entered into a database and analyzed using SPSS 17.0 statistical software. The local progression-free survival rate, distant progression-free survival rate and overall survival rate were summarized using Kaplan–Meier methods. The local progression-free survival was defined as the time interval between the end of CRT and the first observation of local disease progression. Similarly, distant progression-free survival was defined as the time interval between the end of CRT and the first observation of distance metastasis.

## Results

### Patient characteristics

Fifty-one patients with unresectable LRR of breast cancer after (modified) radical mastectomy were enrolled in our monocentric study from January 2008 to June 2012.

Table 1 summarizes the characteristics of the patients at primary diagnosis and prior treatment. The initial staging was as follows (UICC 6th edition): stage I: 9 patients; stage II: 30 patients; and stage III: 12 patients. Primary therapy consisted of radical or modified radical mastectomy in all 51 patients, followed by postoperative chemotherapy (48 patients) and/or hormonal therapy (32 patients). No one received adjuvant HER2-targeted therapy. No one received postmastectomy RT.Patient characteristics at recurrence diagnosis and recurrence treatment are shown in Table 2. The median time from primary surgery to LRR was 24 months (range 3–180 months). A total of 52.9% patients developed recurrence within the first 2 years of primary surgery. The sites of recurrence were confined to CW in 20 patients (32.8%), to SC in 19 (31.1%), to Ax in 11 (18.0%), and IM in the remaining 11 (18.0%). Recurrence was presented as a single nodule in 25 patients, as multiple nodules in 20 patients and as diffuse growth in 6 patients. Radiotherapy fields (CTV1) was as follows: CW + SC&IC only: 18 patients; CW + SC&IC + neck: 11 patients; CW + SC&IC + Ax: 6 patients; CW + SC&IC + IM: 8 patients; CW + SC&IC + neck + IM: 3 patients; CW + SC&IC + neck + Ax: 5 patients. After concurrent RT and paclitaxel, systemic treatment was applied in 42 patients, including chemotherapy alone in 8 patients, hormone therapy alone in 30 patients, sequential chemotherapy and hormone therapy in 2 patients, and HER2-targeted therapy and hormone therapy in 2 patients. After concurrent CRT, no one underwent surgical resection of the LRR.

**Table 1:** Patient characteristics at primary diagnosis and prior treatment

Parameters	No. of patients	Proportion of patients (%)
Age at primary diagnosis		
Median(y)	45	–
Range(y)	31–67	–
≤50 y	36	70.6
>50y	15	29.4
T stage at primary diagnosis		
T1	19	37.3
T2	29	56.9
T3	3	5.9
T4	0	0
Primary N stage		
N0	21	41.2
N1	20	39.2
N2	5	9.8
N3	5	9.8
Primary stage		
I	9	17.6
II	30	58.8
III	12	23.5
ER/PR status at primary diagnosis		
Negative	16	31.4
Positive	33	64.7
Not available	2	3.9
HER-2/neu status at primary diagnosis		
Negative	38	74.5
Positive ^a^	6	11.8
Not available	7	13.7
Prior surgery		
Modified radical mastectomy	42	82.4
Radical mastectomy	9	17.6
Prior chemotherapy		
Yes	48	94.1
No	3	5.9
Prior Hormone therapy		
TAM	27	52.9
AI	5	9.8
No	19	37.3

^a^: Patients were considered to have HER-2/neu positive disease if primary tumor was positive for HER-2 by immunohistochemistry (+++) or by fluorescent in-situ hybridization

**Table 2:** Patient characteristics at recurrence diagnosis and recurrence treatment

Parameters	No. of patients	Proportion of patients (%)
Age at recurrence diagnosis		
Median(y)	49	–
Range(y)	34–69	–
≤50 y	30	58.8
>50y	21	41.2
Disease-free interval		
<1 year	16	31.4
1–2 year	11	21.6
>2 year	24	47.1
Site of recurrence (*n* = 61^a^)		
Chest wall (CW)	20	32.8
Supraclavicular nodes (SC)	19	31.1
Internal mammary lymph nodes (IM)	11	18.0
Axillary lymph nodes (Ax)	11	18.0
No. of recurrence		
Single nodule	25	49.0
Multiple nodules	20	39.2
Diffuse growth	6	11.8
Size of largest recurrence		
<3 cm	32	62.7
≥3 cm	19	37.3
Radiotherapy fields (CTV1) ^c^		
CW + SC&IC only	18	35.3
CW + SC&IC + neck	11	21.6
CW + SC&IC + Ax	6	11.8
CW + SC&IC + IM	8	15.7
CW + SC&IC + neck + IM	3	5.9
CW + SC&IC + neck + Ax	5	9.8
Systemic treatment after concurrent CRT^b^		
None	9	17.6
Chemotherapy	8	15.7
Hormone therapy	30	58.8
Sequential chemotherapy and hormone therapy	2	3.9
HER2-targeted therapy and hormone therapy	2	3.9

^a^: Multiple locoregional recurrences represented 10 patients (3 patients with supraclavicular node and intramammary lymph node; 5 with supraclavicular and axillary lymph node; 1 with supraclavicular node and chest wall; 1 with chest wall and axillary lymph node)

^b^Concurrent CRT: Concurrent radiation therapy and paclitaxel

^c^*CW* chest wall, *SC&IC* supra- and infraclavicular regions, *Ax* axillary lymph node, *IM* intramammary lymph node

### Feasibility of concurrent chemoradiation

In all, 49 patients (96.1%) received assigned doses of RT as scheduled (mean relative dose intensity 99.7%). Two patients required early termination of RT. One experienced severe grade 3 radiation dermatitis. Another experienced both grade 3 radiation dermatitis and grade 3 leukocytopenia. Thirteen patients (25.5%) required a break in the course of RT, with a median delay of 4 days (2–8 days) due to acute toxicity.

Thirty-eight patients (52.1%) received all planned weekly paclitaxel doses. The median paclitaxel cycles actually administered were 5 (range, 2–5) and the mean relative dose intensity was 92.2%.

### Tumor response

We assessed tumor response at 3 months after completion of concurrent RT and paclitaxel (Table 3). Overall, 47 patients (92.2%) achieved clinical response: 36 patients (70.6%) had a complete response (CR) and 11 patients (21.6%) a partial response (PR). Four patients failed to achieve clinical response: 2 (3.9%) had stable disease (SD) and 2 (3.9%) progressive disease (PD). Two patients with PD had tumor regression immediately after completion of concurrent RT and paclitaxel, but new recurrent nodules occurred in the treatment fields at 3 months after completion of concurrent CRT.

**Table 3:** Clinical response rates for locoregional recurrent breast cancer (3 months after completion of concurrent radiation therapy and paclitaxel)

	No. of patients	Proportion of patients (%)
Complete response (CR)	36	70.6
Partial response (PR)	11	21.6
Stable disease (SD)	2	3.9
Progressive disease (PD)	2^a^	3.9

^a^2 patients with progressive disease had tumor regression immediately after completion of concurrent radiation therapy and paclitaxel, but new recurrent nodules occurred in the treatment fields at 3 months after completion of concurrent chemoradiation

### Disease control and survival

**Fig. 1:**
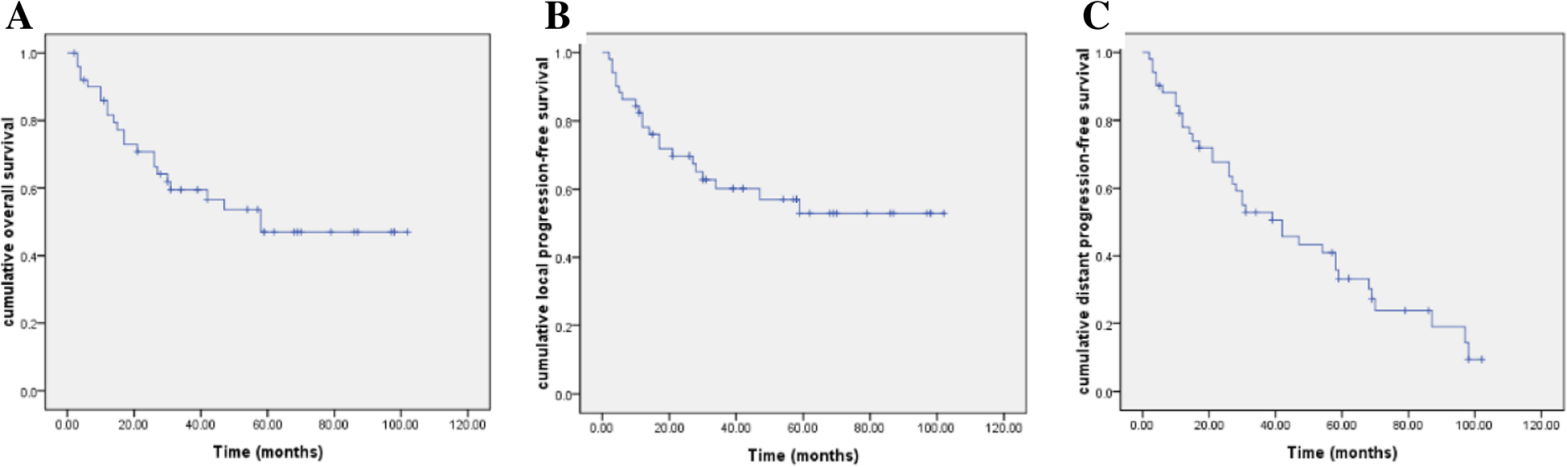
The cumulative survival rate for all patients after completion of chemoradiation. ***a*** *The* cumulative overall survival rate for all patients after completion of chemoradiation, **b** The cumulative local progression-free survival rate, **c** The cumulative distant progression-free survival rate

The median follow-up for all patients after completion of CRT was 42 months (range, 5–111 months). Fourteen patients were lost to follow-up within 5 years. Only 4 patients were lost to follow-up within 30 months. At the time of the last follow-up, local control was achieved clinically in 30 patients (58.9%), and 28 patients had no locoregionally recurrent sites. Fifteen patients (29.4%) developed second recurrences in the treatment fields or field margins, and 6 patients (11.8%) had initial recurrence progression. The cumulative local progression-free survival rate was 62.8% (SE, ± 7.0%) 2 year and 53.0% (SE, ± 8.0%) 5 years after treatment. Thirty-seven patients (72.5%) developed distant metastasis, and the cumulative distant progression-free survival rate was 67.7% (SE, ± 6.7%) 2 year and 33.3% (SE, ± 7.1%) 5 years after treatment. Twenty-three patients (45.1%) had died, and the cumulative overall survival rate was 70.8% (SE, ± 6.6%) 2 year and 47.0% (SE, ± 8.0%) 5 years after treatment (Fig. 1).

### Univariate analysis of prognostic factors

Univariate analysis of prognostic factors related to the 5-year local progression-free survival rate, distant progression-free survival rate and overall survival rate are shown in Table 4. Disease-free interval and number of recurrences were significantly correlated with differences in local progression-free survival, distant progression-free survival and overall survival. The tumor response was significantly correlated with the difference in overall survival.

**Table 4:** Univariate analysis of prognostic factors related to local progression-free survival rate, distant progression-free survival rate and overall survival rate

Parameters	No. of patients	5-year local progression-free survival rate (%)	5-year distant progression-free survival rate (%)	5-year overall survival rate (%)
Age at time of recurrence				
≤50 years	30	50.8 ± 11.3	29.1 ± 8.6	43.3 ± 10.5
>50 years	21	56.8 ± 11.8	48.7 ± 11.8	49.9 ± 12.3
*P* value		0.995	0.476	0.912
Disease-free interval				
≤2 year	27	34.8 ± 11.6	18.7 ± 8.0	25.3 ± 10.0
> 2 year	24	73.2 ± 10.5	57.5 ± 10.9	69.4 ± 10.7
*P* value		0.014	0.007	0.003
No. of recurrence				
Single	25	80.0 ± 8.0	58.8 ± 10.1	73.2 ± 9.5
Multiple and diffused	26	18.2 ± 10.6	10.7 ± 7.0	14.5 ± 8.9
*P* value		0.003	0.001	0.001
Size of largest recurrence				
< 3 cm	32	52.1 ± 10.7	31.0 ± 8.8	40.6 ± 10.3
≥3 cm	19	55.8 ± 13.0	46.2 ± 11.8	53.9 ± 12.2
*P* value		0.819	0.769	0.885
Systemic treatment after concurrent CRT				
Yes	46	59.8 ± 9.1	38.0 ± 8.0	48.5 ± 8.9
No	9	29.6 ± 16.4	29.6 ± 16.4	33.9 ± 18.2
*P* value		0.080	0.453	0.378
Tumor response				
CR	36	60.4 ± 9.4	38.6 ± 8.5	52.9 ± 9.6
PR/SD/PD	15	33.6 ± 15.3	30.2 ± 13.6	27.9 ± 13.3
P value		0.054	0.799	0.047

### Acute toxicity

Table 5 shows the incidence of acute toxicity during concurrent RT and weekly paclitaxel. Neither grade 4 toxicity nor treatment-related deaths occurred. Radiation dermatitis in the treatment field was the most prominent. Grade 3 radiation dermatitis occurred in 10 patients (19.6%). Mucositis with dysphagia occurred frequently, and grade 2 mucositis with dysphagia occurred in 7 patients (13.7%). No one had grade 3 radiation pneumonitis, and only 1 patient (2.0%) experienced grade 2 radiation pneumonitis. Hematological toxicity of grade 3 included leukocytopenia in 12 patients (23.5%). No one had thrombocytopenia.

**Table 5:** Acute toxicity

Toxicity	Grade 1 No. (%)	Grade 2 No. (%)	Grade 3 No. (%)	Grade 4 No. (%)
Hematological				
Leukocytopenia	10 (19.6)	23 (45.1)	12 (23.5)	0 (0)
Thrombocytopenia	0 (0)	0 (0)	0 (0)	0 (0)
Non-hematological				
Radiodermatitis	18 (35.3)	23 (45.1)	10 (19.6)	0 (0)
Pneumonitis	-^a^	1 (2.0)	0 (0)	0 (0)
Mucositis with dysphagia	9 (17.6)	7 (13.7)	0 (0)	0 (0)
Vomiting	1 (2.0)	0 (0)	0 (0)	0 (0)
Cardiac	0 (0)	0 (0)	0 (0)	0 (0)

^a^We did not assess asymptomatic grade 1 radiation pneumonitis because of no conventional chest X-ray or CT film taken in the time of evaluating acute toxicity

## Discussion

To the best of our knowledge, no previous prospective report has evaluated the efficacy and safety of weekly paclitaxel concurrent with RT for the treatment of unresectable locoregionally recurrent breast cancer after mastectomy. This very first prospective study assesses whether the regimen of concurrent RT and weekly paclitaxel is effective and safe in patients with unresectable LRR. The published literature offers limited available data on the optimum choice of chemotherapeutic agents concurrent with RT when treating locoregionally recurrent breast cancer. An overall local response rate of 92.2% was achieved. Grade 3 radiodermatitis and leukocytopenia were observed in 19.6 and 23.5%, respectively. No one had grade 3 radiation pneumonitis, and only 1 patient (2%) experienced grade 2 radiation pneumonitis.

The optimal treatment for recurrent breast cancer is still undefined, especially in non-irradiated patients for their primary disease. Currently, the standard treatment for locoregionally recurrent breast cancer after mastectomy is surgical resection if technically feasible. However, the rate of second local failure with local excision alone remains high [4], and some patients cannot undergo curative surgery because of locally unresectable disease, medical unfitness and an unwillingness to accept the associated complications. To patients with inoperative LRR after mastectomy who had not previously received RT, chest wall and nodal RT has been recommended [17]. The chest wall and supraclavicular region are common re-recurrent sites. It is recommended that the entire chest wall be treated, and field size was proved to be an important factor for freedom from recurrence [6]. Elective radiotherapy of the supraclavicular region in cases of chest wall recurrence is strongly advised. Halverson et al. reported that the supraclavicular failure rate was 16% without elective radiotherapy versus 6% with elective radiotherapy (*p* = 0.0489) for local-regional recurrence following mastectomy [18]. Based on these considerations, the entire chest wall and supraclavicular region were included in the CTV to each patient in our study.

To achieve a better local response, a higher total dose (≥60–70 Gy) normally needs to be delivered to recurrent sites, especially to larger tumors [19]. However, adequate RT doses are difficult to deliver, and toxicity can be significant because of the limited RT tolerance of organs at risk, such as skin and lung. The combination of radiotherapy and chemotherapy can be an attractive option to optimize the doses of RT and the systemic treatment, to reduce their toxicity and to increase the efficacy in terms of local control and metastases. However, the optimal regimens for concurrent CRT are not clearly defined. Preclinical studies have demonstrated the radiosensitizing activity of paclitaxel [9]. The limited available prospective data of phase I/II trials of locally advanced breast cancer and retrospective data on recurrent breast cancer have revealed a benefit of paclitaxel combined with RT and have demonstrated the safety and efficacy of this combination [11, 20]. In these trials, paclitaxel as concurrent chemotherapy was administered in a variety of schedules and doses [11, 12, 20, 21]. Burstein et al. [22] published a Phase I dose–escalation study of 40 Stage II or III breast cancer patients who received concurrent RT with paclitaxel. Dose-limiting toxicity was reached in 4 of 16 patients (25%) who received weekly paclitaxel at 60 mg/m^2^ per week with concurrent RT. In our study, concurrent chemotherapy consisting of weekly paclitaxel at 50 mg/m^2^ for five consecutive weeks (days 1, 8, 15, 22, and 29) was administrated. The regimen of concurrent RT and weekly paclitaxel was effective and safe in patients with LRR in our study.

Our results showed an overall local response rate of 92.2% (complete response 70.6%) at 3 months after completion of concurrent RT and paclitaxel. These findings are comparable to previous retrospective data on recurrent breast cancer and to several available prospective trials of locally advanced breast cancer. Semrau et al. [20] retrospectively analyzed the use of concurrent RT and taxane chemotherapy in patients with LRR of breast cancer. They reported an 89% overall response rate in 27 patients, and no one had PD immediately after completion of CRT. In our study, 2 patients (3.9%) had PD at 3 months after completion of concurrent CRT. However, we should reinforce that the 2 patients with PD had tumor regression immediately after completion of CRT. Several small phase I and II trials investigating the addition of paclitaxel to concurrent CRT in the treatment of locally advanced breast cancer have been reported, and the overall local response rate has been 89–94.9% [21, 23, 24].

In the current study, our local progression-free survival rate of 62.8% 2 year and 53.0% 5 years after treatment are comparable to other studies with CRT or RT alone [20, 25, 26]. Semrau et al. [20] retrospectively reported an 83% local recurrence-free survival rate 1 year and 68% 2 years after concurrent radiotherapy and taxane chemotherapy in patients with inoperable (*n* = 29) or resected (*n* = 7) LRR of breast cancer.

Our results showed that 37 patients (72.5%) had developed distant metastasis by a median of 42 months of follow-up, and the cumulative distant progression-free survival was 67.7% 2 years and 33.3% 5 years after treatment. The patients with distant metastasis had a considerably worse prognosis, in line with previously published data [27, 28]. Systemic therapy supplementary to local treatment is a sound practice. For example, one study recruited 162 patients with LRR breast cancer [7]. After surgery and radiotherapy, the patients were randomly assigned to chemotherapy vs no chemotherapy. The 5-year DFS and OS for the two groups were 69% vs 57% (*P* = 0.046) and 88% vs 76% (*P* = 0.02). More large randomized trials are needed to determine if the substantial risk of systemic relapse following a LRR can be reduced by the administration of chemotherapy either alone or in addition to appropriate hormonal therapy or targeted therapy.

In our study, acute toxicity data showed that the regimen of weekly paclitaxel concurrent with RT was acceptable and manageable in the treatment of LRR in breast cancer. Hematological toxicity of grade 3 included leukocytopenia in 23.5% of patients. Leukocytopenia is not a severe problem, and it can be recovered by granulocyte colony-stimulating factor, followed by completing the chemotherapy.

Radiation dermatitis was more frequent and severe in our study, and grade 3 radiation dermatitis occurred in 10 patients (19.6%). However, our patients received higher RT doses and target volume than others on neoadjuvant or adjuvant therapy [22, 24]. Since the observed radiation dermatitis often occurred near or after the end of RT, it had a minimal effect on the complete treatment. However, patients with grade 3 radiation dermatitis must be monitored carefully and begin supportive treatment in a timely manner.

Symptomatic radiation pneumonitis is perceived as a feared complication. Its incidence after RT only in patients with breast cancer is known to be 1 to 3% [29, 30]. Despite the use of weekly paclitaxel to RT, only 1 patients (2.0%) experienced grade 2 radiation pneumonitis in our study. Semrau et al. [20] reported a symptomatic radiation pneumonitis incidence of 3% (1 out of 36) in recurrent breast cancer patients who received concurrent CRT. However, there are conflicting reports in the literature [22, 31, 32]. In fact, the incidence of radiation pneumonitis is known to be correlated with the functional performance status of the pretreatment lung, the volume of the irradiated lung, the RT dose and the use of concurrent chemotherapy.

One limitation of the current study was that our study was underpowered to detect the hypothesized improvement of LRR, possibly due to the relatively small sample size. Another limitation was that we were not able to obtain enough data about late toxicity. The long-term efficacy and late toxicity could be elucidated after further follow-up.

## Conclusion

Concurrent CRT of weekly paclitaxel with RT exhibited promising efficacy in unresectable locoregionally recurrent breast cancer after mastectomy. Toxicity profiles were acceptable with the dosage and schedule used. Concurrent RT combined with newer cytotoxic and targeted agents may be promising to further improve the treatment of recurrent breast cancer.

## Data Availability

The datasets used and/or analyzed during the current study are available from the corresponding author on reasonable request.
